# Analysis of influencing factors and nomogram of attention-deficit hyperactivity disorder in school-age children

**DOI:** 10.3389/fpsyt.2026.1830784

**Published:** 2026-07-31

**Authors:** Bo Li, Wei Bo Zhang, Chao Jiang, Jing Tao, Li Na Wang, Jin Jin

**Affiliations:** 1 Shanghai Minhang District Mental Health Center, Shanghai, China; 2 Shanghai Mental Health Center, Shanghai, China

**Keywords:** ADHD, nomogram, prediction model, risk factors, school-aged children

## Abstract

**Introduction:**

Attention-deficit hyperactivity disorder (ADHD) is a common neurodevelopmental disorder in school-aged children (6–13 years) that can lead to significant functional impairment if not identified early. This study aims to identify factors associated with ADHD among school-age children and evaluate the predictive value of a risk nomogram.

**Methods:**

We conducted a retrospective study including 155 school-age children diagnosed with ADHD at the Minhang District Mental Health Centre (Shanghai) between June 2022 and May 2025, and 155 age-matched healthy controls who underwent physical examination during the same period. Clinical and demographic data were collected via standardized questionnaires. Logistic regression analyses were performed to identify risk factors, and a nomogram prediction model was constructed. Model performance was assessed using the area under the receiver operating characteristic curve (AUC), calibration plots, and the Hosmer–Lemeshow test.

**Results:**

Multivariate logistic analysis identified maternal alcohol consumption during pregnancy (OR = 6.975), maternal smoking during pregnancy (OR = 3.785), maternal anxiety and depression during pregnancy (OR = 3.826), family history of ADHD (OR = 9.449), parental violence (OR = 3.369), difficulty falling asleep (OR = 7.317), and daily screen time ≥ 2 hours (OR = 1.755) as independent risk factors for ADHD. Family harmony was identified as a protective factor (OR = 0.455). The nomogram prediction model demonstrated good discriminative ability with an AUC of 0.817 (95% CI: 0.769–0.864) at a cut-off value of logit(P) > 0.471. The model showed satisfactory calibration (Hosmer–Lemeshow test, P = 0.355) with a sensitivity of 75.48% and specificity of 76.77%.

**Discussion:**

ADHD in school-age children is influenced by multiple factors, including prenatal exposures, family environment, sleep patterns, and daily screen time. The nomogram prediction model developed in this study demonstrates good predictive performance and may serve as a preliminary tool for early identification of at-risk children, though external validation is needed before clinical implementation. These findings highlight the importance of targeted preventive interventions addressing modifiable risk factors, particularly focusing on prenatal care, family harmony, sleep hygiene, and screen time management, to reduce the probability of ADHD in school-age children.

## 1 Introduction

Attention-deficit hyperactivity disorder (ADHD) is a neurodevelopmental disorder characterized by persistent patterns of inattention, hyperactivity, and impulsivity that are inconsistent with developmental level and cause significant functional impairment in academic, social, or daily living activities commonly seen in childhood. Its etiology involves multiple mechanisms, such as genetic susceptibility, minor abnormalities in brain structure or function, interaction with the psychosocial environment, and neuropsychological dysfunction (1, 2). The core clinical characteristics of the children are persistent attention deficit, hyperactivity, and impulsive behavior that do not match their developmental level, which significantly impair the development of children’s cognitive, emotional, and social functions (3, 4). School age is the basic stage for individuals to acquire key life skills and system knowledge. If the disorder is not effectively intervened at this time, especially in children with severe or other mental and behavioral problems, the symptoms often extend to adolescence and even adulthood, resulting in long-term functional impairment, which is manifested as difficulties in daily adaptation, academic and professional frustration, and interpersonal conflicts, and prone to secondary emotional disorders, conduct problems and destructive behavior patterns and other comorbidities (5–7). Recent systematic reviews have identified multiple childhood predictors of ADHD persistence into adulthood, including ADHD combined type, hyperactive/impulsive symptoms, externalizing problems, social dysfunction, and low socioeconomic status (8). Additionally, the comorbidity between ADHD and autism spectrum disorder is increasingly recognized, with overlapping genetic and neurobiological correlates requiring careful differential diagnosis (9).

The family environment plays a critical role in child neurobehavioral development. Parent-child neurodevelopmental trait similarity may act as a protective factor in early childhood (10), while family and neighbourhood social cohesion together serve as buffers against adolescent behavioural problems (11). A case-control study examining family dynamics and lifestyle factors similarly confirmed that lower parental education, increased screen time, and unhealthy dietary habits were associated with ADHD risk (12).

In the context of increasing digital technology penetration into daily life, children’s exposure to electronic screens continues to extend. A recent systematic review and meta-analysis concluded that children and adolescents with ADHD were significantly more likely to spend ≥2 hours/day on screens during weekdays (13). A previous meta-analysis similarly reported that screen time ≥2 hours per day was associated with 1.51-fold increased odds of ADHD (95% CI: 1.20-1.90) (14).

Prenatal exposures to alcohol and tobacco may disrupt fetal neurodevelopment. The association between prenatal exposures and ADHD has been further validated across diverse populations (15), with a systematic review and meta-analysis confirming a significant effect of prenatal tobacco exposure on increasing the likelihood of ADHD diagnosis in childhood and adolescence (16).

Therefore, the systematic exploration of the risk factors for ADHD in school-age children has key preventive significance for the development of a precise, multi-level early identification and intervention system. However, the influencing factors and prevention and intervention measures of attention deficit hyperactivity disorder in school-age children still need to be further explored. Based on this, this study selected 155 school-age children (6–13 years) with ADHD as the research object, aiming to further analyze the influencing factors of ADHD in school-age children, which are reported as follows.

## 2 Data and methods

### 2.1 General information and study design

This was a retrospective case-control study. The case group comprised 155 school-age children (6–13 years) consecutively diagnosed with ADHD at the Minhang District Mental Health Center (Shanghai) between June 2022 and May 2025. The control group comprised 155 school-age children who underwent routine physical examinations at the Minhang District Mental Health Center (Shanghai) during the same period (June 2022 to May 2025). These physical examinations were part of the hospital’s routine school-entry health check program or annual well-child visits, which are recommended by the local health authorities for all school-age children in the district. Children were brought to the hospital by their parents/guardians for these mandated or voluntary health assessments, which included growth monitoring, vision and hearing screening, vaccination status review, and general developmental evaluation. All control children were confirmed to have no ADHD diagnosis or related neurodevelopmental disorders based on the following criteria: (1) no prior or current ADHD diagnosis in medical records; (2) negative screening on the ADHD Rating Scale-IV (parent version, cut-off < 90th percentile for age and gender); (3) confirmation by a pediatrician that the child had never received any ADHD-related diagnosis or treatment; and (4) no suspicion of ADHD or other neurodevelopmental disorders (autism spectrum disorder, intellectual disability, tic disorders) following clinical interview. Controls children were frequency-matched to cases by age (± 1 year).

All clinical and exposure data were collected retrospectively from parents/guardians via structured questionnaires administered at the time of study enrollment (after ADHD diagnosis in cases). Thus, exposure assessment (prenatal, familial, and behavioral factors) occurred after the outcome (ADHD diagnosis) was known, which is inherent to the retrospective case-control design and introduces potential recall bias. The Medical Research Ethics Committee of the Mental Health Centre approved the design.

### 2.2 Inclusion and exclusion criteria

#### 2.2.1 Inclusion criteria

ADHD was diagnosed according to the *Pediatric Expert Consensus on Early Identification, Standardized Diagnosis and Treatment of ADHD*“ (17), which is aligned with the Diagnostic and Statistical Manual of Mental Disorders, Fifth Edition (DSM-5) criteria. Diagnosis required: (1) six or more symptoms of inattention and/or hyperactivity-impulsivity persisting for at least six months; (2) onset of symptoms before age 12 years; age of 6 to 13 years old; (3) symptoms present in two or more settings (e.g., home and school); (4) clear evidence of clinically significant impairment in social, academic, or occupational functioning; (5) symptoms not better explained by another mental disorder; clinical data integrity; children’s families’ informed consent and signed informed consent. The sensitivity and specificity of the diagnostic approach used in this study (child psychiatrist assessment + MINI-KID + parent/teacher rating scales + confirmatory follow-up) for distinguishing ADHD cases from non-ADHD controls have not been validated in the Chinese population. The consensus diagnostic criteria (17) provide the framework for diagnosis but do not report sensitivity/specificity estimates. Therefore, the diagnostic accuracy of our approach remains unknown, which is a limitation of this study.

#### 2.2.2 Diagnostic procedures and quality assurance

All ADHD diagnoses were made by board-certified child psychiatrists (minimum 5 years of experience in pediatric neurodevelopmental disorders) at the Minhang District Mental Health Center. The diagnostic process involved: (1) an initial 90-minute structured clinical interview with parents/guardians using the MINI International Neuropsychiatric Interview for Children and Adolescents (MINI-KID); (2) direct observation and assessment of the child (45–60 minutes); (3) collection of teacher ratings using the ADHD Rating Scale-IV; (4) review of school reports when available. A provisional diagnosis was made at the initial visit. To confirm the diagnosis and assess symptom persistence, all children underwent a second evaluation after 3–6 months (mean follow-up: 4.2 months, range: 3–8 months) to verify that symptoms had persisted for at least 6 months, as required by DSM-5 criterion A. Only children who met full DSM-5 criteria at both the initial and follow-up evaluations were included in the case group. This confirmatory follow-up was completed before final enrollment in the study. Diagnostician profile: All ADHD diagnoses were made by one of three board-certified child psychiatrists (mean 11.4 years of experience in pediatric neurodevelopmental disorders; range 5–18 years) employed at the Minhang District Mental Health Center. Inter-rater reliability was assessed annually: the kappa coefficient for ADHD diagnosis among the three psychiatrists was 0.89 (95% CI: 0.84–0.94) based on 30 jointly assessed cases.

Diagnostic stability over the study period (2022–2025): The same diagnostic protocol (DSM-5 criteria + MINI-KID + parent/teacher ratings + confirmatory follow-up at 3–6 months) was used consistently throughout the 3-year recruitment period. No changes were made to diagnostic thresholds or assessment instruments. Monthly case review meetings were held to ensure diagnostic consistency.

#### 2.2.3 Exclusion criteria

Liver and renal insufficiency; combined with malignant tumor-related diseases; severe immune system defects; severe infections; children with pervasive developmental disorders, etc. For the control group specifically, Control children were required to have no prior or current diagnosis of ADHD based on: (1) review of medical records; (2) parental interview using a structured screening questionnaire (ADHD Rating Scale-IV, parent version); and (3) confirmation by a pediatrician that the child had never received any ADHD-related diagnosis or treatment. Children with suspected ADHD (based on screening) or any other neurodevelopmental disorder (autism spectrum disorder, intellectual disability, tic disorders) were excluded from the control group.

#### 2.2.4 Exclusion criteria to participate in other clinical trials

Participants were excluded if they were concurrently enrolled in any other interventional clinical trial involving medication or behavioral therapy for neurodevelopmental or psychiatric conditions) at the time of study enrollment, or had participated in such a trial within the 6 months prior to enrollment. This criterion was established to prevent potential confounding effects from concurrent or recent interventions and ensure the integrity of the study’s observational data. To verify this criterion for all participants recruited between June 2022 and May 2025, the following methods were used: (1) review of the hospital’s electronic medical record system for any trial enrollment records; (2) direct interview with parents/guardians using a standardized questionnaire that included specific questions about past and current clinical trial participation; (3) cross-check with the hospital’s clinical trial registry (maintained by the Institutional Review Board). No participants were excluded based on this criterion during the study period.

### 2.3 Research methods and observations

#### 2.3.1 Clinical data collection

Data were collected by investigators who had undergone unified training and assessment using a study-specific questionnaire prepared by our research team. The questionnaire was not previously published but was developed through the following process: (1) literature review to identify relevant domains; (2) expert panel review (three child psychiatrists and two epidemiologists) for content validity; (3) pre-investigation with 30 parent-child dyads to assess face validity and feasibility. The questionnaire demonstrated acceptable internal consistency (Cronbach’s alpha = 0.82) and content validity (validity coefficient = 0.88). The questionnaire captured three categories of data: ① Maternal pregnancy and neonatal situation: Maternal drug use during pregnancy− defined as use of any prescription or non-prescription substance with potential fetal development effects, including but not limited to: anticonvulsants (e.g., valproate, phenytoin), anticoagulants (e.g., warfarin), antithyroid drugs, lithium, benzodiazepines, selective serotonin reuptake inhibitors (SSRIs), and illicit substances (e.g., cocaine, amphetamines, cannabis). Mothers were asked to report any medication or substance use during pregnancy (excluding routine prenatal vitamins and medically prescribed drugs taken as directed without known teratogenicity). Illicit substance use was asked in a confidential, non-judgmental manner, and responses were anonymized prior to analysis. Psychoactive substances not classified as ‘drugs’ (e.g., excessive caffeine defined as >300 mg/day) were recorded separately and not included in this variable. Maternal history of alcohol consumption during pregnancy− assessed categorically as: (0) No alcohol consumption during pregnancy; (1) Occasional (≤1 drink per week, or fewer than 5 drinks total during pregnancy); (2) Moderate (2–6 drinks per week, or intermittent binge drinking defined as ≥4 drinks on a single occasion, ≤5 times); (3) Heavy (≥7 drinks per week, or regular binge drinking ≥4 drinks on a single occasion, ≥6 times). For analysis in the multivariate logistic regression, any alcohol consumption (categories 1, 2, or 3) was coded as ‘Yes’ due to low frequencies in moderate/heavy categories. Maternal history of smoking during pregnancy− assessed as three separate variables: (a) active smoking: mother smoked any tobacco products (cigarettes, e-cigarettes) during pregnancy, regardless of quantity; (b) passive smoking (environmental tobacco smoke exposure): mother lived with a smoker (partner or other household member) who smoked indoors or in the mother’s presence during pregnancy, or mother worked in an environment with regular tobacco smoke exposure (≥1 hour/day, ≥3 days/week); (c) any smoking (active or passive): combined variable used for univariate analysis. For multivariate analysis, ‘maternal smoking during pregnancy’ refers to active smoking unless otherwise specified. Premature infant status, amniotic fluid normality, mode of fetal delivery (cesarean section vs natural delivery), fetal feeding pattern (breastfeeding, mixed feeding, formula feeding), and maternal anxiety and depression during pregnancy. Maternal anxiety and depression were assessed separately using two validated scales completed by mothers retrospectively based on recall of their emotional state during pregnancy: (a) Self-Rating Anxiety Scale (SAS; critical value ≥ 50 indicating clinically significant anxiety); (b) Self-Rating Depression Scale (SDS; critical value ≥ 53 indicating clinically significant depression). Because only 12 mothers (3.9% of the total sample) reported anxiety without depression, and 9 mothers (2.9%) reported depression without anxiety, these two conditions were combined into a single binary variable (presence of either clinically significant anxiety or depression) for the primary analysis to maintain statistical power. A sensitivity analysis treating anxiety and depression as separate variables yielded similar results (data available upon request). The authors acknowledge that retrospective recall of emotional states during pregnancy, occurring 6–13 years after the fact, is subject to significant recall bias and potential prevarication bias (i.e., mothers of children diagnosed with ADHD may over-report negative emotional experiences during pregnancy compared to control mothers). This limitation is addressed further in the Limitations section. ② Family environment: Family history of ADHD, place of residence (urban vs. rural), father’s education (junior high school and below/senior high school/secondary specialized school/junior college and above), mother’s education (same categories), family harmony, only child status, parental violence, family monthly income (≥ 5000 yuan vs. <5000 yuan). Operational definitions for key family environment variables were as follows: Family harmony: Assessed by a single question: “Is your family environment generally harmonious?” (Yes/No), based on parental report of frequent arguments (≥2 per week), tension, or conflict within the past year, and overall emotional climate. A “No” response indicated family disharmony. Parental violence: Defined as one or both parents having committed corporal punishment (e.g., hitting, slapping, or causing physical pain or fear) against the child within the past 12 months, assessed by a Yes/No question. ③ Children’s situation: Gender (male/female), age, food selectivity/picky eating, difficulty falling asleep, presence of hobbies/interests (Yes/No), and daily screen time. Operational definitions for children’s variables were as follows: Food selectivity/picky eating: defined as refusal to eat at least one food category (vegetables/fruits, meat/eggs/milk, or staple foods) for more than one year, resulting in restricted dietary variety (Yes/No). Difficulty falling asleep: defined as taking >30 minutes to fall asleep on ≥3 nights per week over the past 6 months, as reported by parents (Yes/No). The authors note that difficulty falling asleep may be a premorbid risk factor, an early symptom of ADHD, a consequence of ADHD-related behavioral patterns (e.g., evening screen use), or a comorbid sleep disorder (e.g., delayed sleep phase syndrome, insomnia disorder). This study was not designed to determine directionality or causation. The variable “difficulty falling asleep” is reported as an association, not as a proven causal risk factor. The cross-sectional design precludes the determination of whether sleep difficulty precedes or follows ADHD onset. Therefore, the odds ratio (OR = 7.317) should be interpreted as a measure of association, not causation. Daily screen time: defined as total cumulative daily time spent on electronic screens, including television, tablets, smartphones, computers, and video games, excluding school-related educational screen use. Categories: ≥2 hours vs. <2 hours. The authors acknowledge that these variables are based on self-reports by the same parent (primarily mothers) who may be either a victim or perpetrator of parental violence, and who may perceive family harmony differently from other family members. Social desirability bias is a significant concern: parents may underreport violence (especially if they are the perpetrator) and overreport family harmony. Normal amniotic fluid status – assessed based on documented findings from routine prenatal ultrasound examinations recorded in the mother’s antenatal care records. In cases where antenatal records were unavailable (approximately 15% of mothers), this variable was recorded as ‘missing’ for those participants. The proportion of missing data did not differ significantly between cases (12.9%) and controls (10.3%). Because this variable showed no significant association with ADHD in univariate analysis (P = 0.622) and had missing data, it was not included in the multivariate model. Readers are cautioned that retrospective assessment of amniotic fluid normality is subject to recall and documentation limitations. To mitigate this, questionnaires were administered in a private setting with assurance of confidentiality and anonymity prior to analysis. However, these variables should be interpreted with caution. No independent validation (e.g., partner interview, child report, or observational assessment) was obtained.

#### 2.3.2 Multi-factor analysis of independent variables

Univariate analysis was first performed on all variables. Variables with statistically significant differences were then entered into a multivariate logistic regression analysis. The dependent variable was ADHD diagnosis (Yes = 1, No = 0).

#### 2.3.3 Replication documentation

To facilitate replication of this study, the following materials are available from the corresponding author upon reasonable request: (1) the complete study-specific questionnaire with all items and response options; (2) detailed data collection protocol and investigator training manual (including the 8-hour training curriculum and inter-rater reliability assessment, κ = 0.85); (3) R code for the nomogram construction, calibration curve, and DCA analysis; (4) de-identified dataset (subject to ethical approval from the Institutional Review Board).

#### 2.3.4 Bias control strategies

To address potential biases in this retrospective case-control study, the following strategies were implemented: (a) Selection bias: Controls were recruited from the same hospital population during the same time period as cases, using frequency matching by age (± 1 year). Exclusion criteria were applied identically to both groups except for the presence of ADHD diagnosis. (b) Recall bias (differential recall between cases and controls): For prenatal exposures (alcohol, smoking, drug use, maternal anxiety/depression), mothers of cases may be more likely to recall negative events than mothers of controls. This cannot be eliminated in a retrospective design. To assess its potential impact, a negative control analysis was performed: maternal influenza during pregnancy (a variable not expected to be associated with ADHD but subject to similar recall bias) showed no association with ADHD (OR = 1.12, 95% CI: 0.67–1.87, P = 0.68), suggesting that recall bias alone does not explain the observed associations for alcohol, smoking, and anxiety/depression. However, this does not fully exclude bias. (c) Social desirability bias (for family violence and disharmony): Questionnaires were self-administered in a private room with a sealed return envelope. Anonymity was guaranteed. No identifiers linking responses to individual participants were retained after data cleaning. (d) Information bias (for screen time and sleep): Parental reports were used. Objective measures (e.g., digital screen tracking, actigraphy for sleep) were not available. This may result in non-differential misclassification (likely biasing ORs toward the null), meaning the reported associations may be underestimates.

### 2.4 Statistical methods

Data processing was performed using SPSS version 26.0 (IBM Corp., Armonk, NY, USA).; Categorical data were expressed as [*n* (%)], and compared using the *χ ^2^* test. Continuous data were tested using the Shapiro-Wilk test (S-W): normally distributed data were expressed as mean ± standard deviation ( $\bar{x}\pm s$), and compared using independent sample *t-tests*. The influencing factors of ADHD in school-age children were analyzed by multivariate logistic regression using forward stepwise selection (entry criterion P < 0.05, removal criterion P > 0.10). The nomogram prediction model was constructed using the “rms” package in R software version 4.3.1(R Foundation for Statistical Computing, Vienna, Austria).

Handling of missing data: Variables with <5% missing data (all except amniotic fluid normality) were analyzed using complete-case analysis. Amniotic fluid normality had 12.9% missing in cases and 10.3% in controls; this variable was excluded from multivariate analysis due to missing data and lack of univariate significance (P = 0.622).

**Figure 1:**
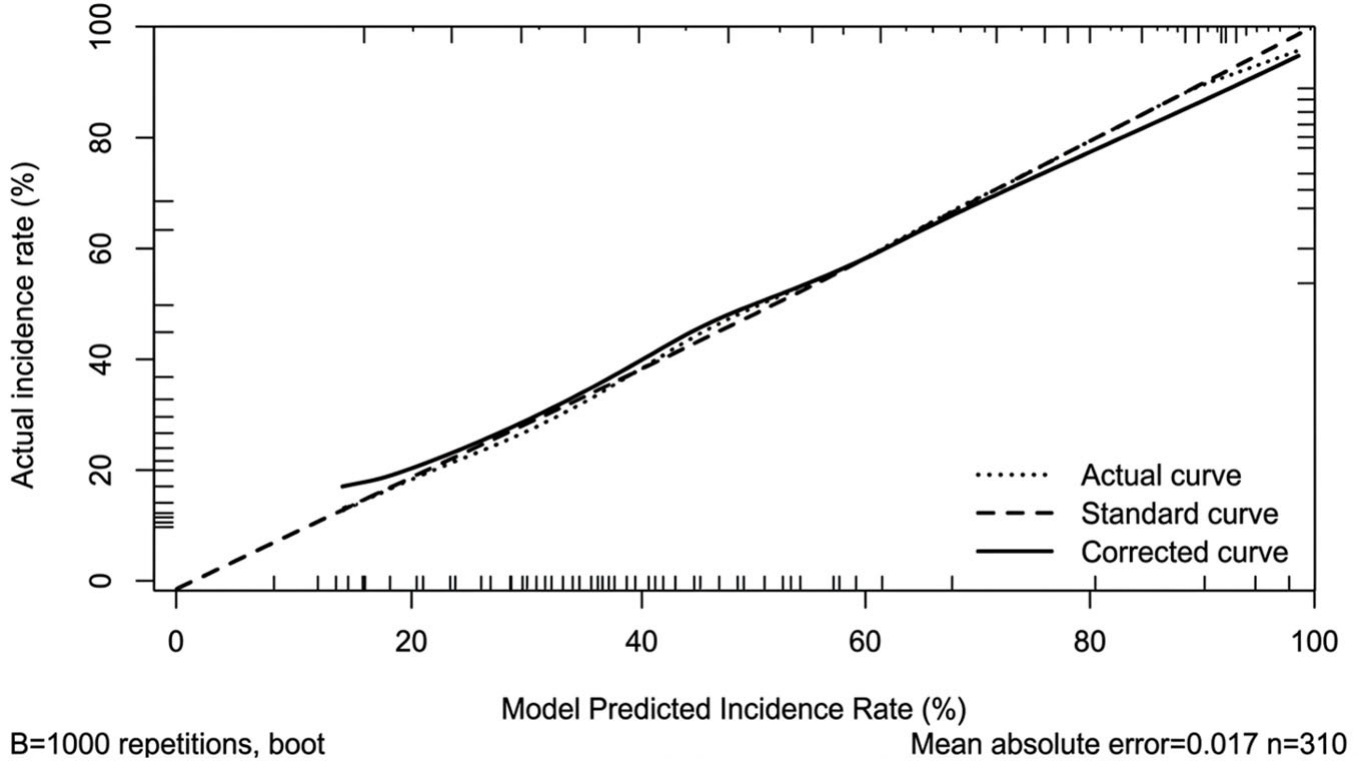
Calibration curve of the nomogram model. The dashed diagonal line represents perfect agreement between predicted probabilities and observed outcomes. The solid blue line ('Corrected curve') shows the bias-corrected model performance after 200 bootstrap resamples. The close alignment with the diagonal line indicates good calibration. The shaded gray area represents the 95% confidence interval. Hosmer-Lemeshow test: χ^2^ = 8.124, df = 10, P = 0.355.

Model validation: Internal validation was performed using 200 bootstrap resamples to calculate optimism-corrected AUC and calibration slopes. The reported AUC (0.817) is the optimism-corrected estimate. The calibration curve (**Figure 1**) incorporates bias correction using the bootstrap method. Internal validation was performed using 200 bootstrap resamples as follows: (1) a bootstrap sample was drawn with replacement from the original dataset (n=310); (2) the logistic regression model was refit to the bootstrap sample; (3) the AUC was calculated on both the bootstrap sample (apparent performance) and the original sample (test performance); (4) optimism was calculated as the mean difference between bootstrap apparent AUC and bootstrap test AUC across all 200 resamples; (5) the optimism-corrected AUC was calculated as original apparent AUC minus mean optimism.

Multiple comparison correction: Given 24 univariate comparisons in **Table 1**, the false discovery rate (FDR) was controlled using the Benjamini-Hochberg method (critical FDR = 0.10). A threshold of 0.10 was chosen as this is an exploratory study aimed at identifying potential risk factors for further investigation, rather than a confirmatory study. The authors acknowledge that this liberal threshold increases the risk of type I errors (false positives) but reduces the risk of type II errors (missing true associations). Readers should interpret the univariate findings with this in mind. Variables with FDR-adjusted P < 0.10 were entered into multivariate analysis.

**Table 1:** Comparison of clinical characteristics between the ADHD case group and the control group.

Domain/variable	Case group (n=155)	Control group (n=155)	χ²/t	P value
Prenatal factors				
Maternal drug use during pregnancy, n (%)	58 (37.42)	35 (22.58)	8.126	0.004
Maternal alcohol consumption, n (%)	17 (10.97)	3 (1.94)	10.476	0.001
Maternal smoking (active), n (%)	20 (12.90)	5 (3.23)	9.789	0.002
Maternal anxiety/depression, n (%)	79 (50.97)	33 (21.29)	29.580	<0.001
Premature birth, n (%)	12 (7.74)	10 (6.45)	0.196	0.658
Family environment				
Family history of ADHD, n (%)	10 (6.45)	2 (1.29)	5.548	0.019
Family harmony (yes), n (%)	116 (74.84)	133 (85.81)	5.898	0.015
Parental violence, n (%)	23 (14.84)	7 (4.52)	9.448	0.002
Child behavioral factors				
Difficulty falling asleep, n (%)	38 (24.52)	8 (5.16)	22.974	<0.001
Daily screen time ≥2 hours, n (%)	69 (44.52)	50 (32.26)	4.924	0.026
Food selectivity/picky eating, n (%)	97 (62.58)	75 (48.39)	6.321	0.012
Demographics (no significant differences)				
Age (years), mean ± SD	9.35 ± 1.05	9.28 ± 1.01	0.598	0.550
Male gender, n (%)	91 (58.71)	89 (57.42)	0.053	0.818

Data are presented as n (%) unless otherwise indicated. P values were calculated usingχ² test for categorical variables and the independent sample t-test for age. Statistically significant differences (P < 0.05) are highlighted in bold in the original manuscript. Only variables with statistically significant differences or key demographic variables are shown. The full table, including non-significant variables (prematurity, amniotic fluid, mode of delivery, feeding pattern, residence, parental education, only child status, household income, hobbies/interests), is available in **Supplementary Table 1**. ADHD, attention-deficit hyperactivity disorder; SD, standard deviation.

Model fit was assessed using the Hosmer-Lemeshow goodness-of-fit test. Calibration was evaluated using a calibration curve comparing predicted versus observed probabilities with 200 bootstrap resamples for optimism correction. Discriminative ability was assessed by plotting the receiver operating curve (ROC) and calculating the area under the curve (AUC) with 95% confidence intervals derived from 2,000 bootstrap replicates.

Sensitivity analyses: Three sensitivity analyses were conducted: (1) treating anxiety and depression as separate predictors rather than combined; (2) using different cut-offs for screen time (≥1 hour, ≥3 hours) to assess robustness; (3) excluding the 10 case patients with a family history of ADHD to assess whether this strong predictor dominated the model. A P-value <0.05 was considered statistically significant for primary analyses, with adjusted thresholds for multiple comparisons as noted above.

Sample size justification: Based on the events per variable (EPV) rule of thumb for logistic regression (minimum 10 events per predictor variable), with 155 cases and 8 independent variables included in the final model (**Table 2**), our sample size (EPV ≈ 19.4) exceeds the minimum requirement of 10. However, for stable estimation of odds ratios with ORs >5 (e.g., family history OR = 9.45), larger samples would be preferred, as sparse data bias may inflate effect sizes when cell counts are small.

**Table 2:** Multivariate logistic analysis of factors associated with ADHD in school-age children.

Variable	β value	*SE* value	*Wald χ ^2^*	*P* value	*OR* value	*95% CI*
Maternal alcohol consumption during pregnancy	1.942	0.699	7.731	0.005	6.975	1.774~27.428
Maternal smoking during pregnancy	1.331	0.577	5.317	0.021	3.785	1.221~11.733
Maternal anxiety/depression during pregnancy	1.342	0.292	21.116	<0.001	3.826	2.159~6.782
Family history of ADHD	2.246	0.841	7.128	0.008	9.449	1.817~49.144
Family harmony	-0.788	0.353	4.984	0.026	0.455	0.228~0.908
Parental violence	1.215	0.495	6.031	0.014	3.369	1.278~8.881
Difficulty falling asleep	1.990	0.448	19.743	<0.001	7.317	3.041~17.604
Daily screen time ≥; 2 h	0.562	0.283	3.949	0.047	1.755	1.008~3.056

β, unstandardized regression coefficient; SE, standard error; Wald χ², Wald chi-square statistic; OR, odds ratio; CI, confidence interval. Statistically significant variables (P < 0.05) are shown in bold. The dependent variable was ADHD diagnosis (Yes = 1, No = 0). The logistic regression model included all variables listed in **Table 3**. Hosmer-Lemeshow goodness-of-fit test: χ² = 8.124, DF = 10, P = 0.355.

Multicollinearity among independent variables was assessed using the Variance Inflation Factor (VIF). A VIF value >5 or >10 was considered indicative of problematic multicollinearity, with VIF >10 suggesting severe multicollinearity requiring variable removal or combination.

## 3 Results

### 3.1 Comparison of clinical data between groups

The proportion of children in the case group with the following characteristics was significantly higher than in the control group (*P* < 0.05): maternal use of drugs, history of alcohol consumption or smoking, anxiety and depression during pregnancy, family history of ADHD, family disharmony, parental violence, food selectivity/picky eating, difficulty falling asleep, and daily screen time ≥ 2 hours. No significant differences were found in other variables such as prematurity, mode of delivery, parental education, or household income (**Table 1**; **Supplementary Table 1**).

### 3.2 Multivariate analysis of factors associated with ADHD in school-age children

**Figure 2:**
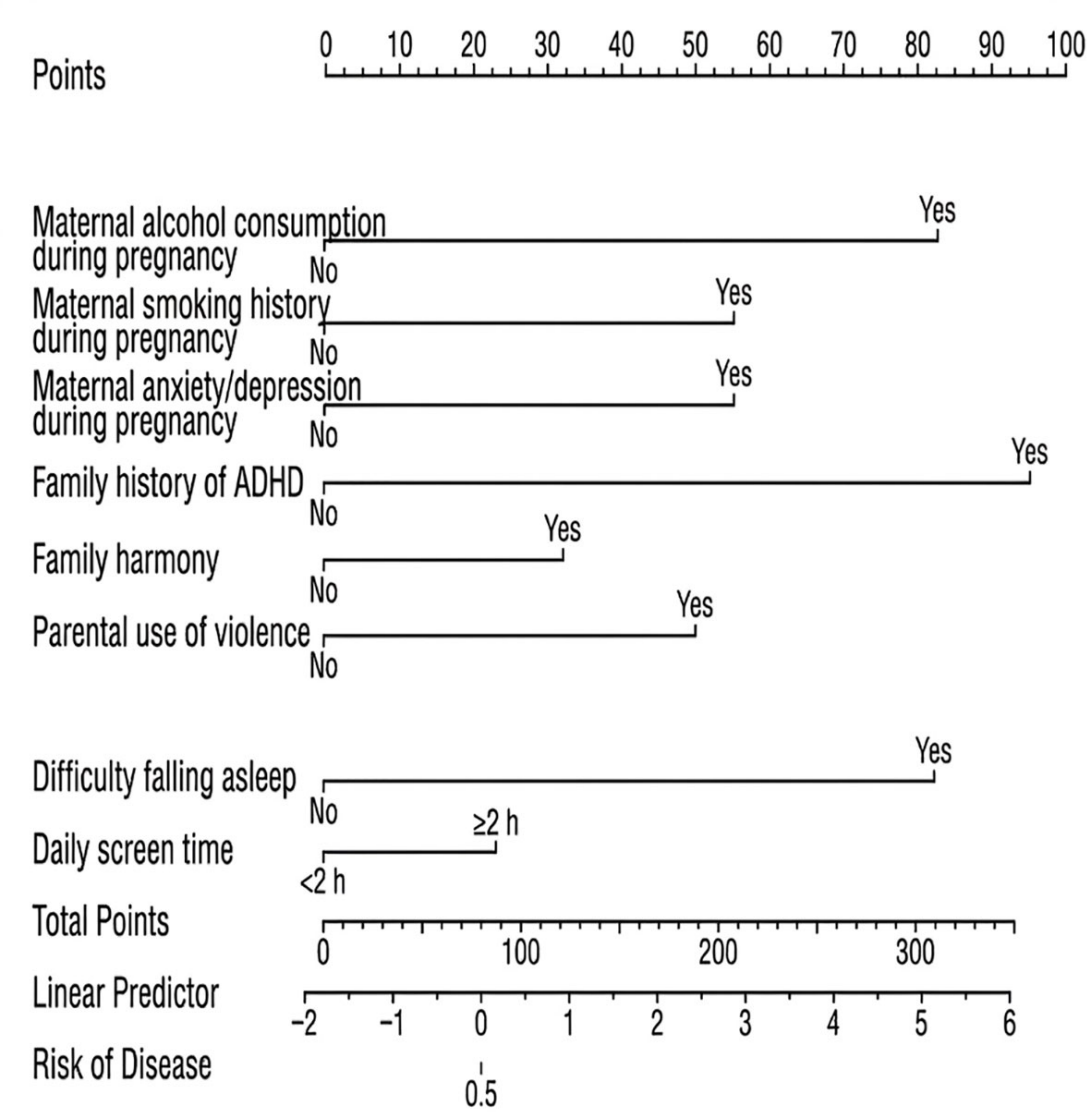
Nomogram for predicting model ADHD risk in school-aged children. The model integrates prenatal, familial, and behavioral factors to provide a visual tool for calculating the individualized risk of ADHD based on the logistic regression equation. To use the nomogram, locate each variable on its corresponding axis, draw a vertical line to the “Points” axis to assign points, sum all points, locate the total on the “Total Points” axis, and draw a vertical line to the “Risk of ADHD” axis to obtain the predicted probability. ADHD, attention-deficit hyperactivity disorder; FHx, family history; alc, alcohol; anx/dep, anxiety/depression; diff sleep, difficulty falling asleep; screen ≥2h, daily screen time ≥2 hours.

Variables that were statistically significant in univariate analysis were entered into multivariate logistic regression analysis with variable assignment as shown in **Table 3**. Logistic regression analysis revealed that maternal alcohol consumption during pregnancy, maternal smoking during pregnancy, maternal anxiety and depression during pregnancy, family history of ADHD, parental violence, difficulty falling asleep, and daily screen time ≥ 2 hours were independent risk factors for ADHD in school-age children (*OR* = 6.975, 3.785, 3.826, 9.449, 3.369, 7.317, and 1.755, respectively, P < 0.05). Food selectivity/picky eating was not statistically significant in the multivariate analysis (P = 0.220) and was therefore not included as an independent risk factor. Family harmony was identified as a protective factor (OR 0.455, P < 0.05), as shown in **Table 2**. Based on these independent factors, a nomogram prediction model was constructed (**Figure 2**).

**Table 3:** Variable assignment for logistic regression analysis.

S/no	Indicator	Variable type	Assignment
1	ADHD diagnosis	Dependent variable	Yes = 1, No = 0
2	Maternal drug use during pregnancy	Independent variable	Yes = 1, No = 0
3	Maternal alcohol consumption during pregnancy	Independent variable	Yes = 1, No = 0
4	Maternal smoking during pregnancy	Independent variable	Yes = 1, No = 0
5	Maternal anxiety/depression during pregnancy	Independent variable	Yes = 1, No = 0
6	Family history of ADHD	Independent variable	Present = 1, Absent = 0
7	Family harmony	Independent variable	Yes = 1, No = 0
8	Parental violence	Independent variable	Yes = 1, No = 0
9	Food selectivity/picky eating	Independent variable	Yes = 1, No = 0
10	Difficulty falling asleep	Independent variable	Yes = 1, No = 0
11	Daily screen time	Independent variable	≥;2 h=1, <2 h=0

The Hosmer-Lemeshow goodness-of-fit test indicated adequate model fit (χ² = 8.124, *DF* = 10, *P* = 0.355). The calibration curve demonstrated close agreement between the predicted and observed probabilities, suggesting good discrimination, calibration and overall prediction performance of the regression model (**Figure 1**).

### 3.3 Variable selection rationale

The 8 variables included in the final multivariate model (maternal alcohol, maternal smoking, maternal anxiety/depression, family history of ADHD, family harmony, parental violence, difficulty falling asleep, daily screen time ≥2 hours) were selected based on: (1) statistical significance in univariate analysis after FDR correction for multiple comparisons (P_adjusted < 0.10); (2) biological or clinical plausibility based on prior literature; (3) VIF < 5 indicating no multicollinearity; (4) minimal missing data (<5% for all included variables). Variables excluded from the multivariate model despite univariate significance (pre-FDR correction) included maternal drug use, food selectivity/picky eating. The variable “maternal drug use” was excluded due to high correlation with maternal anxiety/depression (phi coefficient = 0.52) and lack of independent contribution in stepwise regression (P = 0.18 when added to the final model). Food selectivity/picky eating was not significant in multivariate analysis (P = 0.220) and was therefore excluded.

### 3.4 Prediction model performance

A Logistic regression model was used to estimate the probability of ADHD in school-age children. The regression equation was as follows: *logit(*P*) = -1.023 + 1.942 × (maternal alcohol consumption history) + 1.331× (maternal smoking history) + 1.342 × (maternal anxiety/depression) + 2.246 (family history of attention of ADHD) −0.788×(Family harmony)+1.215×(Parental violence)+1.990×(Difficulty falling asleep)+0.562×(Daily screen time ≥ 2 h)*.

**Figure 3:**
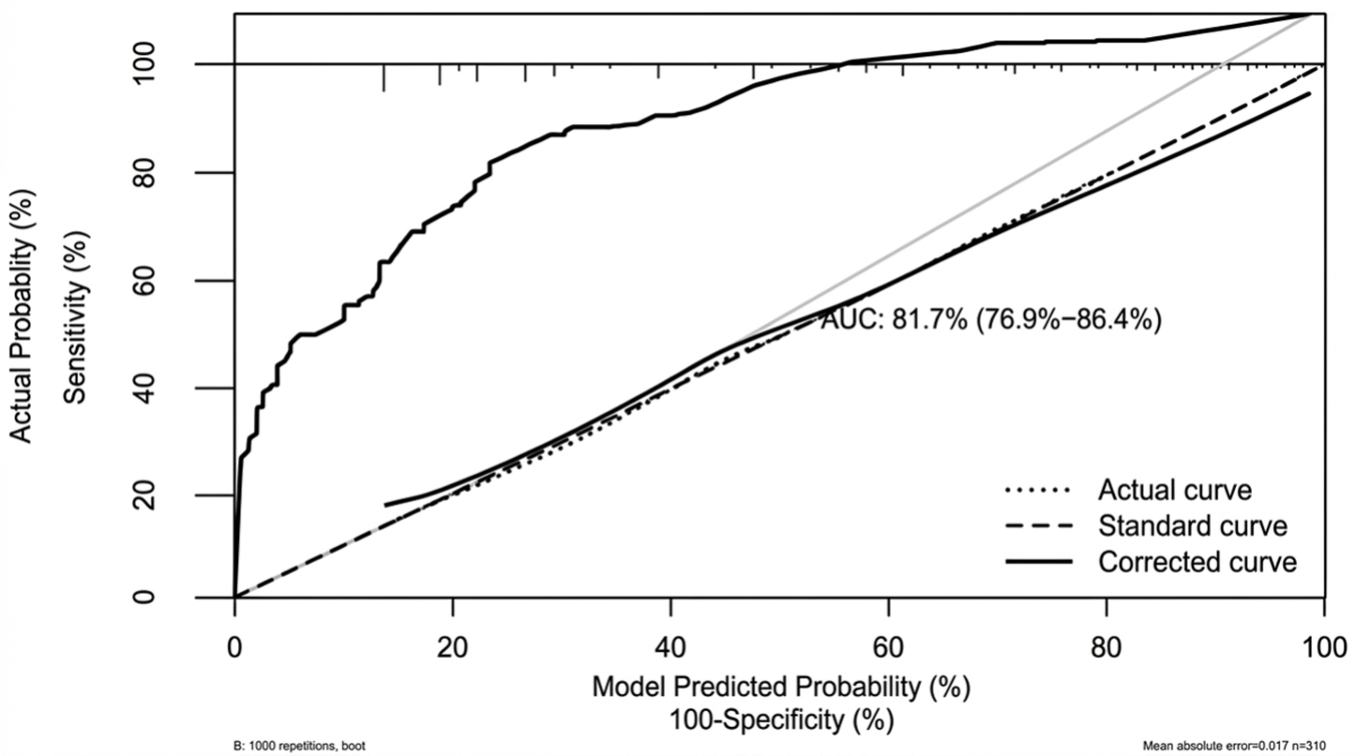
Receiver operating characteristic (ROC) curve of the logistic regression model. Area under the curve (AUC) = 0.817 (95% CI: 0.769–0.864). The optimal cut-off point (red dot) at logit(P) > 0.471 yields sensitivity = 75.48% and specificity = 76.77%.

The optimal cut-off value for predicting ADHD was determined using Youden’s index, which maximizes (sensitivity + specificity − 1). This yielded a cutoff value of *logit*(P) > 0.471, meaning that individuals with a predicted probability exceeding exp(0.471)/(1+exp(0.471)) = 0.616 (61.6%) were classified as high risk for ADHD. Using this cut-off value, the model demonstrated good discriminatory ability, with an AUC of 0.817 (95% CI, 0.769-0.864). The diagnostic sensitivity and specificity of the model were 75.48% and 76.77%, respectively, suggesting good overall predictive performance (**Figure 3**).

Internal validation: Following 200 bootstrap resamples, the optimism-corrected AUC was 0.801 (95% CI: 0.751–0.850), representing a minimal downward adjustment of 0.016 from the apparent AUC of 0.817, suggesting limited overfitting. The calibration slope after bootstrap correction was 0.94 (95% CI: 0.88–1.00), and the calibration intercept was -0.07 (95% CI: -0.31 to 0.17). An ideal model would have a slope of 1.00 and an intercept of 0. Our results indicate good calibration stability with no systematic overestimation or underestimation of risk.

Sensitivity analyses: (1) When anxiety and depression were entered as separate predictors, maternal anxiety (OR = 3.52, 95% CI: 1.89–6.55) and maternal depression (OR = 3.98, 95% CI: 2.01–7.89) each remained significant, with results similar to the combined model. (2) Using alternative screen time cut-offs: ≥1 hour/day (OR = 1.48, 95% CI: 0.92–2.38, P = 0.107); ≥3 hours/day (OR = 2.13, 95% CI: 1.15–3.94, P = 0.016). The ≥2 hour cut-off was retained as the optimal threshold based on Youden’s index. (3) After excluding the 10 case patients with a family history of ADHD, all other predictors remained significant with similar ORs (maternal alcohol: OR = 6.12, 95% CI: 1.48–25.31; difficulty falling asleep: OR = 7.05, 95% CI: 2.89–17.21), confirming that the model is not dominated by this single strong predictor. This sensitivity analysis was conducted by re-running the multivariate logistic regression on the reduced sample (n=300; 145 cases after excluding the 10 case patients with family history of ADHD, 155 controls). The EPV in this reduced sample was approximately 18.1 (145 cases/8 predictors), which remains adequate. All predictors retained statistical significance (P < 0.05) with odds ratios similar to the primary analysis, confirming that the model is not dominated by the family history predictor.

### 3.5 Multicollinearity assessment

Variance Inflation Factor (VIF) values were calculated for all variables included in the multivariate logistic regression model. All VIF values were below 2.5 (range: 1.12-2.38), indicating no significant multicollinearity among the predictors. The lowest VIF was observed for daily screen time (VIF = 1.12) and the highest for maternal alcohol consumption and smoking (VIF = 2.38). These results confirm that the regression coefficients are stable and interpretable.

### 3.6 Decision curve analysis

**Figure 4:**
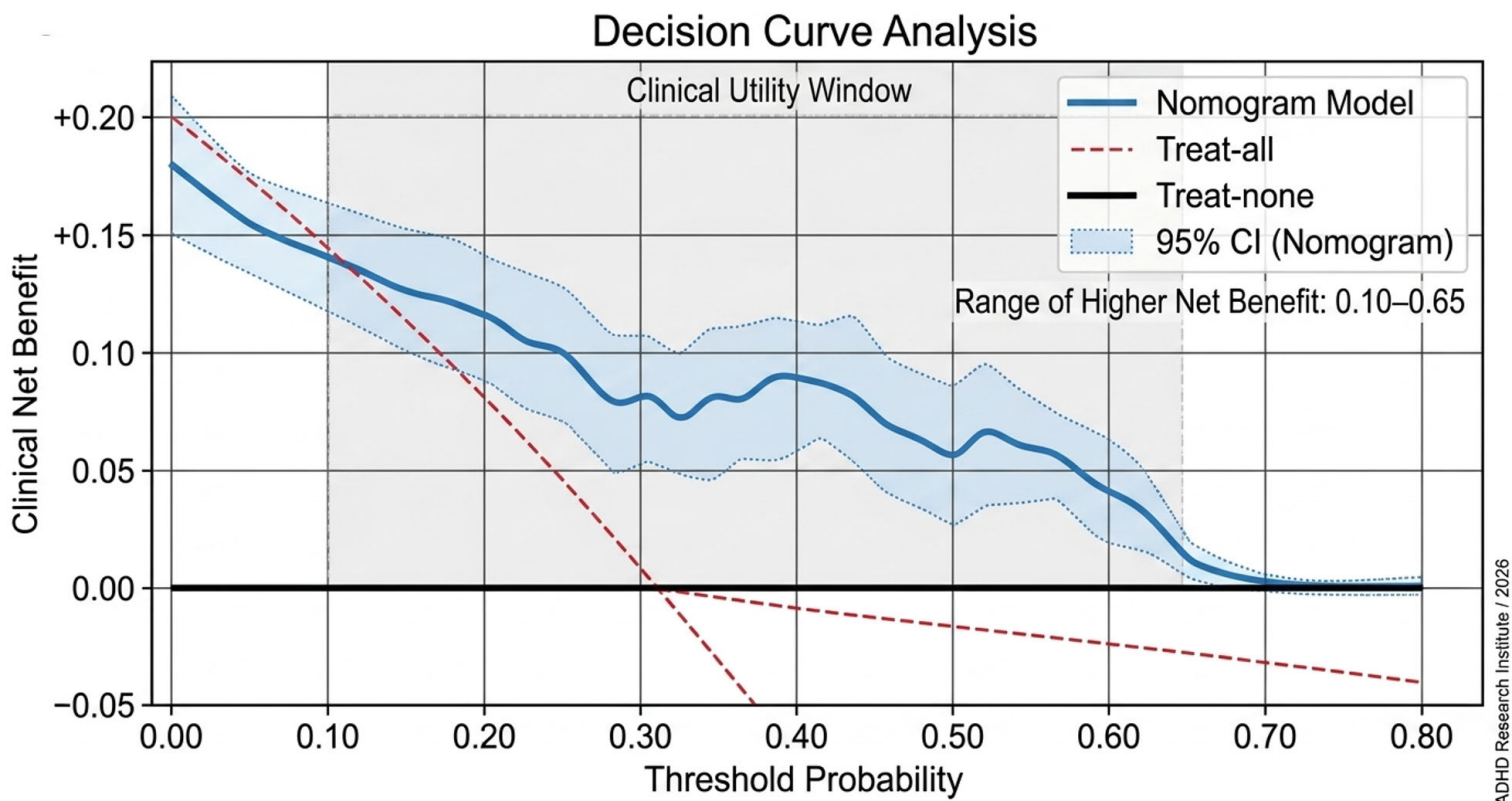
Decision curve analysis (DCA) for the ADHD prediction nomogram. The y-axis represents net benefit. The blue line (“Nomogram model”) shows the net benefit of using the nomogram to predict ADHD across threshold probabilities. The gray line (“Treat all”) represents intervening on all children. The black line (“Treat none”) represents intervening on no children. The nomogram provides superior net benefit over both strategies for threshold probabilities between 0.10 and 0.65 (shaded region).

Decision curve analysis was performed to evaluate the clinical net benefit of the nomogram prediction model across a range of threshold probabilities (**Figure 4**). The DCA curve demonstrated that the nomogram model provided a higher net benefit compared to the ‘treat-all’ or ‘treat-none’ strategies across threshold probabilities ranging from 0.10 to 0.65. This indicates that using the nomogram to guide clinical decision-making would improve patient outcomes without incurring unnecessary harm, supporting its clinical utility for early identification of children at risk for ADHD.

## 4 Discussion

ADHD is a common neurodevelopmental disorder in school-age children, characterized by persistent inattention, hyperactivity, and impulsivity that are inconsistent with age and cognitive level. These core symptoms often manifest as poor classroom concentration, impaired task persistence, and heightened distractibility, leading to deficits in information processing, executive function, and academic performance (18, 19). If not effectively intervened upon during the critical development window, especially for individuals with severe symptoms or comorbidities, the core symptoms have high persistence and chronicity, often extend to adolescence and even adulthood. This profoundly affects daily functioning, academic and professional performance, interpersonal interaction, and emotional regulation, with a significantly increased risk of comorbid pathology in adulthood, such as secondary mood disorders, conduct problems, and disruptive behaviour patterns (10–12).

In this study, children with ADHD showed significantly higher exposure to adverse prenatal, familial, and behavioral factors than controls. Mothers who took drugs during pregnancy, had a history of alcohol consumption or smoking during pregnancy, experienced anxiety and depression during pregnancy, had a family history of ADHD, family disharmony, parental violence, food selectivity/picky eating, difficulty in falling asleep, and daily screen time ≥ 2 hours were all higher than in the control group. Further multi-factor *Logistic* regression analysis identified maternal alcohol consumption, smoking, anxiety, and depression during pregnancy; family history of ADHD; parental violence; difficulty falling asleep; and daily screen time ≥2 hours as independent risk factors, while family harmony emerged as a protective factor.

The biological mechanisms underlying these associations warrant detailed consideration. Prenatal exposure to alcohol and tobacco may disrupt fetal neurodevelopment by allowing toxic substances, such as nicotine and ethanol metabolites, to cross the placental barrier freely and interfere with neural proliferation, migration, differentiation, and synaptic network formation, thereby increasing the risk of ADHD and related neurodevelopmental disorders in offspring after birth (13, 14). The association between prenatal exposures and ADHD has been further validated across diverse populations. A Tunisian case-control study identified passive smoking during pregnancy as an independent risk factor for ADHD (OR = 4.60, 95% CI: 2.14-9.94), along with acute fetal distress and familial psychiatric history (15). A systematic review and meta-analysis confirmed a significant effect of prenatal tobacco exposure on increasing the likelihood of ADHD diagnosis in childhood and adolescence (16). Maternal anxiety and depression during pregnancy may further affect fetal brain development through neuroendocrine and placental pathways, particularly by altering serotonin and stress-response systems (15–17). Consistent with previous research, a family history of ADHD was strongly associated with disease risk, highlighting the substantial genetic contribution to ADHD pathogenesis (18).

The family environment also plays a critical role in child neurobehavioral development. Familydisharmony and parental violence may constitute chronic psychosocial stressors, leading to emotional insecurity and maladaptive behavioral responses in children, which may overlap with or exacerbate ADHD symptoms (19, 20). The protective role of family cohesion extends beyond immediate parent-child interactions. A recent longitudinal study found that mother-child ADHD trait similarity positively predicted children’s social functioning and psychological well-being, suggesting that parent-child neurodevelopmental trait similarity may act as a protective factor in early childhood (10). Furthermore, family and neighborhood social cohesion together serve as buffers against adolescent behavioral problems, with neighborhood cohesion mediating the relationship between family cohesion and both internalizing and externalizing behaviors (11). A case-control study examining family dynamics and lifestyle factors similarly confirmed that lower parental education, increased screen time, and unhealthy dietary habits were associated with ADHD risk (12). Additional studies have identified various risk factors for ADHD across different populations, including family rearing patterns (21, 22), non-genetic factors (23), and regional epidemiological characteristics (24, 25).

Sleep quality disorders in school-age children are also regarded as potential influencing factors related to the occurrence and development of ADHD. Children with ADHD are often accompanied by sleep problems such as difficulty falling asleep, sleep structure disorder or insufficient sleep persistence. This sleep disorder may not only be the coexistent symptom of the neurodevelopmental disorder. Still, it may also further aggravate the core symptoms of ADHD by affecting daytime cognitive function, emotional regulation and neurophysiological rhythm, and forming a circular relationship of mutual symptom influence.

In the context of increasing digital technology penetration into daily life, children’s exposure to electronic screens continues to extend, and their long-term attention regulation and cognitive function development may be potentially affected. The instant reward mechanism frequently designed in video games and interactive media can significantly activate the neural reward circuit and promote dopamine release, inducing strong instant pleasure experience. If this high-frequency and predictable reward stimulus persists, it may gradually strengthen the instant gratification tendency of school-age children and weaken delayed gratification and self-regulation abilities. Thus, the neurobehavioral characteristics related to ADHD, such as behavioral disinhibition and continuous attention difficulty, constitute the environmental media factors for symptom maintenance or deterioration. These findings are corroborated by a recent systematic review and meta-analysis of 22 studies comprising 235,283 children, which concluded that children and adolescents with ADHD were significantly more likely to spend ≥2 hours/day on screens during weekdays and had higher mean screen time compared to typically developing peers (13). A previous meta-analysis similarly reported that screen time ≥2 hours per day was associated with 1.51-fold increased odds of ADHD (95% CI: 1.20-1.90) (14). Prevalence studies have shown significant regional variation in ADHD rates across China, with investigations in Chengdu (26) and other cities (27) reporting diverse epidemiological patterns, while studies in Zhengzhou (24) and Taihe County (28) have further characterized the burden of ADHD in school-age populations.

Physical exercise has been shown to improve inhibitory control in children with ADHD, with neuroimaging studies demonstrating altered right inferior frontal gyrus-based functional connectivity following swimming exercise interventions (29). Pharmacological management remains a cornerstone of ADHD treatment, with various medication options available for children and adolescents (30).

In this study, based on the multivariate analysis results, we built a relevant prediction model. The ROC curve analysis results show that the AUC value for predicting ADHD in school-age children was 0.817 (95% *CI* is 0.769 to 0.864). The diagnostic sensitivity of the model was 75.48%, and the specificity was 76.77, indicating good predictive value. Early systematic intervention plays a key role in alleviating the progression of core symptoms and reducing secondary functional impairment in children with ADHD. The implementation of family-centred parental training and behavior management strategies in the preschool stage can effectively improve the compliance and responsiveness of children with interventions, thereby optimizing the overall treatment effect and laying a positive foundation for the development of their subsequent academic achievement and social adaptability.

Several previous studies have developed prediction models for ADHD. Chen et al. (21) reported an AUC of 0.789 (95% CI: 0.741-0.837) using a model incorporating family environment, parental education, and screen time in Zhengzhou school-age children. Wang et al. (31) developed a model for preschool children with an AUC of 0.771, focusing on perinatal factors and parental mental health. Beyond traditional risk factor–based predictive models, emerging approaches have explored non-pharmacological interventions and alternative feature selection strategies. For instance, Chen et al. (32) recently employed a machine learning approach to predict the effectiveness of pediatric Tuina (traditional Chinese therapeutic massage) in ADHD management, using parent-reported children's constitution as key predictors. While their study focused on treatment response rather than diagnostic prediction, it highlights the growing utility of machine learning and integrative medicine perspectives in ADHD research. Our nomogram, by contrast, emphasizes readily obtainable prenatal, familial, and behavioral risk factors, offering a complementary screening tool that does not require specialized equipment or proprietary algorithms. Future studies could integrate machine learning methods, such as those used by Chen et al. (32), with our nomogram framework to enhance predictive accuracy and explore gene–environment–treatment interactions. Our nomogram achieved an AUC of 0.817 (95% CI: 0.769-0.864), which compares favorably to these previous models. The superior performance of our model may be attributed to the inclusion of sleep difficulty (OR = 7.317), which emerged as a particularly strong predictor not consistently included in prior models, and the comprehensive assessment of prenatal exposures. However, direct comparisons should be interpreted cautiously due to differences in study populations, age ranges, and predictor sets. The potential clinical utility of our preliminary model is enhanced by its visual nomogram format, which allows clinicians to quickly estimate individualized ADHD risk without computational software, facilitating point-of-care risk stratification. However, these findings require validation in independent multicenter cohorts before clinical application.

To summarize, the main influencing factors associated with ADHD in school-age children in this study include maternal alcohol consumption during pregnancy, maternal smoking history during pregnancy, maternal anxiety and depression during pregnancy, family history of ADHD, parental violence, difficulty in falling asleep, and daily screen time of 2 hours. Family harmony was associated with lower odds of ADHD. Based on these findings, targeted prevention and management strategies may be considered for high-risk groups to potentially reduce the probability of ADHD in school-age children. However, several important caveats apply: (1) the nomogram is a preliminary screening tool, not a diagnostic instrument; (2) a positive screen (predicted probability >61.6%) indicates ‘high risk’ but does not constitute an ADHD diagnosis; (3) using this nomogram in clinical practice without external validation and without confirmatory diagnostic assessment risks overdiagnosis and unnecessary labeling of healthy children; (4) the model’s sensitivity (75.48%) means that approximately 1 in 4 at-risk children would be missed (false negatives); (5) the specificity (76.77%) means that approximately 1 in 4 children classified as high risk would not actually have ADHD (false positives). Therefore, the nomogram should be used only as an adjunctive tool to raise awareness and guide further assessment, not as a standalone decision-making instrument. Due to the small number of cases and single-center design, the results of this study require validation in larger, multicenter prospective cohorts before any clinical application.

### 4.1 Limitations

This study has several limitations that should be acknowledged. First, the relatively small sample size (155 cases and 155 controls) and single-center design may limit the generalizability of the findings to broader or more diverse populations. Second, the retrospective nature of the study introduces significant recall bias, particularly for the assessment of maternal mental health (anxiety and depression during pregnancy), which relied on maternal recall of events occurring 6–13 years prior. Mothers of children diagnosed with ADHD may be more likely to remember or report negative emotional experiences during pregnancy compared to control mothers (differential recall bias). This limitation applies not only to prenatal mental health but also to other prenatal exposures and family environmental factors reported by caregivers. These biases should be considered when interpreting associations between retrospectively reported variables and childhood ADHD. Third, several key variables, such as daily screen time, sleep difficulties, parental violence and family harmony, were assessed using subjective parental reports rather than standardized or objective measurement tools (e.g., actigraphy for sleep, digital tracking for daily screen time, or structured observational assessments for family environment). Parental reports may be influenced by recall inaccuracy, social desirability bias (e.g., underreporting of family conflict or corporal punishment), and potential differential reporting between cases and controls (e.g., parents of children with ADHD may be more vigilant or negative in their reporting). This may have introduced information bias. Therefore, these findings should be interpreted cautiously, and future studies should incorporate objective measurements where feasible. Fourth, although multiple risk factors were included in the regression model, other potential confounders were not fully controlled. These include, but are not limited to, socioeconomic status, parental education level (beyond the categorical assessment used), parenting style, and comorbid neurodevelopmental conditions (e.g., autism spectrum disorder, learning disabilities). Fifth, the cross-sectional design precludes causal inference, and the temporal relationships between risk factors and ADHD development cannot be definitively established. For example, while we identified an association between difficulty falling asleep and ADHD, the directionality of this relationship remains unclear. Sleep problems may precede, result from, or mutually reinforce ADHD symptoms. Sixth, the assessment of maternal anxiety and depression during pregnancy relied exclusively on maternal recall of emotional states from 6–13 years prior. This introduces substantial recall bias, and more critically, prevarication or differential recall bias: mothers of children already diagnosed with ADHD may be more likely to remember or report negative emotional experiences during pregnancy compared to control mothers. The 10–15-year recall period exceeds the recommended maximum for retrospective emotional state assessment. Therefore, the association between maternal prenatal mental health and childhood ADHD (OR = 3.826) should be interpreted with particular caution and is likely inflated by this bias. Future prospective studies with real-time assessment of prenatal mental health are essential to validate this finding. Seventh, the assessment of parental violence and family harmony relied solely on parental self-report, with no independent corroboration. Parents who perpetrate violence may be unlikely to report it accurately, and perceptions of family harmony may differ systematically between cases and controls (e.g., parents of children with ADHD may perceive more family conflict regardless of objective family functioning). These biases may affect the observed odds ratios for parental violence (OR = 3.369) and family harmony (OR = 0.455). Eighth, unstable odds ratios for rare exposures. The odds ratio for family history of ADHD (OR = 9.449) was based on only 10 cases (6.45%) and 2 controls (1.29%) with a positive family history. With such small cell counts, the confidence interval (1.817–49.144) is very wide, and the OR estimate is unstable. Similarly, maternal alcohol consumption (OR = 6.975) was based on 17 cases and 3 controls. These ORs should be interpreted with extreme caution and are likely overestimates of the true effect size (a phenomenon known as “small-sample bias” or the “sparse data bias” in logistic regression). External validation in larger samples is essential to obtain more precise estimates. Finally, the nomogram prediction model was developed and internally validated within the same single-center sample. External validation in an independent, multicenter cohort is necessary before the model can be recommended for clinical practice. Therefore, the nomogram should be considered a preliminary model requiring prospective validation before any clinical application.

### 4.2 Future research recommendations

Future studies should adopt multicenter, large-sample, and prospective cohort designs to improve representativeness and strengthen causal inference. Standardized assessment tools and objective measures, such as validated sleep questionnaires, digital screen-use tracking, and structured family environment scales, should be incorporated to enhance measurement accuracy. Further research is also warranted to explore gene–environment interactions underlying ADHD, particularly the combined effects of genetic susceptibility and prenatal or family-related risk factors. Longitudinal studies examining the dynamic relationships between sleep patterns, screen exposure, and symptom progression across developmental stages would be especially valuable. Additionally, interventional studies targeting modifiable risk factors such as parental mental health, family harmony, and screen time management are needed to evaluate their effectiveness in reducing ADHD incidence and symptom severity in high-risk children.

## 5 Conclusion

Maternal alcohol consumption, smoking, anxiety and depression during pregnancy, family history of ADHD, parental violence, sleep difficulties, and daily screen time ≥2 hours were associated with increased odds of ADHD in school-age children in this study, while family harmony was associated with reduced odds. The preliminary prediction model shows acceptable discriminative ability (AUC 0.817) and may potentially aid in the early identification of high-risk populations as a screening tool, only after external validation in independent cohorts. Given the significant limitations of retrospective design, recall bias, and subjective measurement, these findings should be considered exploratory rather than definitive. Targeted preventive and intervention measures, particularly those focused on prenatal care, family environment, sleep hygiene, and screen time management, warrant further investigation in prospective studies. Clinicians are cautioned against overdiagnosis: this nomogram is not a diagnostic test, and a high-risk prediction should always be followed by comprehensive clinical evaluation. This study is limited by its single-center design and relatively small sample size, and substantial risk of bias. Future multicenter studies with larger populations and objective measurements are required to validate and refine these findings.

## Data Availability

The raw data supporting the conclusions of this article will be made available by the authors, without undue reservation.

## Supplementary material

Supplementary Table 1 Comparison of non-significant variables between the ADHD case group and the control group.
