# Supplementary material for: Analysis of influencing factors and nomogram of attention-deficit hyperactivity disorder in school-age children
Source: Front Psychiatry. 2026 Jul 31;17:1830784. doi: 10.3389/fpsyt.2026.1830784 (PMC13473084; doi:10.3389/fpsyt.2026.1830784)
Supplement: Supplementary file 1 [file Table1.docx]

Supplementary Table 1: Comparison of clinical data of groups

| Factors | Case group (155 cases) | Control group (155 cases) | *t* /value of *χ ^2^* | *P* value |
| --- | --- | --- | --- | --- |
| Medications taken by mothers during pregnancy [*n* (%)] | - | - | 8.126 | 0.004 |
| Yes | 58(37.42) | 35(22.58) | - | - |
| No | 97(62.58) | 120(77.42) | - | - |
| Maternal history of alcohol consumption during pregnancy [*n* (%)] | - | - | 10.476 | 0.001 |
| Yes | 17(10.97) | 3(1.94) | - | - |
| No | 138(89.03) | 152(98.06) | - | - |
| Maternal history of smoking during pregnancy [*n* (%)] | - | - | 9.789 | 0.002 |
| Yes | 20(12.90) | 5(3.23) | - | - |
| No | 135(87.10) | 150(96.77) | - | - |
| Premature [*n* (%)] | - | - | 0.196 | 0.658 |
| Yes | 12(7.74) | 10(6.45) | - | - |
| No | 143(92.26) | 145(93.55) | - | - |
| Normal amniotic fluid [*n* (%)] | - | - | 0.243 | 0.622 |
| Yes | 132(85.16) | 135(87.10) | - | - |
| No | 23(14.84) | 20(12.90) | - | - |
| Mode of fetal delivery [*n* (%)] | - | - | 0.117 | 0.732 |
| Cesarean section | 73(47.10) | 70(45.16) | - | - |
| natural birth | 82(52.90) | 85(54.84) | - | - |
| Fetal feeding pattern [*n* (%)] | - | - | 2.404 | 0.301 |
| Breastfeeding | 13(8.39) | 19(12.26) | - | - |
| mixed feeding | 65(41.94) | 71(45.81) | - | - |
| milk powder feeding | 77(49.68) | 65(41.94) | - | - |
| Maternal anxiety and depression during pregnancy [*n* (%)] | - | - | 29.580 | <0.001 |
| Yes | 79(50.97) | 33(21.29) | - | - |
| No | 76(49.03) | 122(78.71) | - | - |
| Family history of attention deficit hyperactivity disorder [*n* (%)] | - | - | 5.548 | 0.019 |
| There are | 10(6.45) | 2(1.29) | - | - |
| None | 145(93.55) | 153(98.71) | - | - |
| Residence [*n* (%)] | - | - | 0.232 | 0.630 |
| Town | 105(67.74) | 101(65.16) | - | - |
| Rural | 50(32.26) | 54(34.84) | - | - |
| Father's education [*n* (%)] | - | - | 1.004 | 0.605 |
| Junior high school and below | 68(43.87) | 62(40.00) | - | - |
| Senior high school/Secondary specialized school | 44(28.39) | 42(27.10) | - | - |
| College and above | 43(27.74) | 51(32.90) | - | - |
| Mother's education [*n* (%)] | - | - | 3.946 | 0.139 |
| Junior high school and below | 55(35.48) | 39(25.16) | - | - |
| Senior high school/Secondary specialized school | 66(42.58) | 78(50.32) | - | - |
| College and above | 34(21.94) | 38(24.52) | - | - |
| Family Harmony [*n* (%)] | - | - | 5.898 | 0.015 |
| Yes | 116(74.84) | 133(85.81) | - | - |
| No | 39(25.16) | 22(14.19) | - | - |
| Only child [*n* (%)] | - | - | 0.341 | 0.559 |
| Yes | 93(60.00) | 98(63.23) | - | - |
| No | 62(40.00) | 57(36.77) | - | - |
| Use of violence by parents [*n* (%)] | - | - | 9.448 | 0.002 |
| Yes | 23(14.84) | 7(4.52) | - | - |
| No | 132(85.16) | 148(95.48) | - | - |
| Monthly household income [*n* (%)] | - | - | 0.188 | 0.664 |
| ≥; 5000 yuan | 124(80.00) | 127(81.94) | - | - |
| <5000 yuan | 31(20.00) | 28(18.06) | - | - |
| Gender [*n* (%)] | - | - | 0.053 | 0.818 |
| Male | 91(58.71) | 89(57.42) | - | - |
| Female | 64(41.29) | 66(42.58) | - | - |
| Age, year ($\bar{x}\pm s$) | 9.35±1.05 | 9.28±1.01 | 0.598 | 0.550 |
| Presence of partial eclipse [*n* (%)] | - | - | 6.321 | 0.012 |
| Yes | 97(62.58) | 75(48.39) | - | - |
| No | 58(37.42) | 80(51.61) | - | - |
| Difficulty falling asleep [*n* (%)] | - | - | 22.974 | <0.001 |
| Yes | 38(24.52) | 8(5.16) | - | - |
| No | 117(75.48) | 147(94.84) | - | - |
| Interests [*n* (%)] | - | - | 0.632 | 0.426 |
| Yes | 75(48.39) | 82(52.90) | - | - |
| No | 80(51.61) | 73(47.10) | - | - |
| Daily Video Duration [*n* (%)] | - | - | 4.924 | 0.026 |
| ≥;2 h | 69(44.52) | 50(32.26) | - | - |
| <2 h | 86(55.48) | 105(67.74) | - | - |
